# Pituitary Apoplexy in Geriatric Patients: A Report of Four Cases

**DOI:** 10.7759/cureus.20318

**Published:** 2021-12-10

**Authors:** Meryem Drissi Oudghiri, Imane Motaib, Saloua Elamari, Soukaina Laidi, Asmaa Chadli

**Affiliations:** 1 Department of Endocrinology, Diabetology, Nutrition, and Metabolic Diseases, Shaikh Khalifa Bin Zayed Al-Nahyan Hospital – Mohammed VI University of Health Sciences, Casablanca, MAR

**Keywords:** ante-pituitary insufficiency, surgery, elderly, pituitary macroadenoma, pituitary apoplexy

## Abstract

Pituitary apoplexy (PA) is a rare clinical syndrome related to abrupt hemorrhage and/or infarction of the pituitary gland, usually occurring in patients with preexisting pituitary disease. It is an endocrine emergency requiring rapid diagnosis and appropriate management.

This is a literature review and a retrospective study reporting the observation of four patients that have suffered from pituitary macroadenomas.

These observations illustrate the particularities of this pathology in the elderly. The symptoms may be truncated and lead to a late diagnosis with its repercussions on management, without forgetting the particularity of the fragile and multisystemic terrain, which may contraindicate the usual surgical treatment. A rapid diagnosis and appropriate management can limit the occurrence of irreversible complications.

## Introduction

Pituitary apoplexy (PA) is a sporadic pathology consequent to abrupt hemorrhage, necrosis, or infarction of the pituitary gland secondary to a hypophyseal tumor that in certain cases has a high mortality rate [1]. The pituitary adenoma’s fast evolution constrains the nearby paraspinal anatomical tissues and is usually presented as an acute tumor syndrome with cranial nerve palsy, cephalalgia, ocular disturbances, and endocrine anomalies. This stroke may also reveal the existence of a pituitary tumor. PA may also appear in patients without pituitary tumors, such as in obstetric cases when a surge in the pituitary gland dimensions is observed [2].

To the best of our knowledge, only a few clinical series and literature reviews have been described in the literature investigating the management of PA, and no randomized controlled trials have been described to put together a set of guidelines for the ideal management of this disorder [3]. The management of patients suffering from PA depends on expert opinion findings and selected case series. There are several controversies in the management algorithm for this uncommon endocrine emergency, not to forget whether the conservative treatment or surgical management can be adopted as first-line treatment management and the ideal timing of surgery can be suggested for such cases especially in the elderly for whom the presence of comorbidities, essentially cardiovascular issues, can change the prognosis.

The aim of this study is to detail our experience with this rare disease among elderly patients. We collected retrospectively four observations of elderly patients with pituitary apoplexy. Consent was obtained from all participants.

## Case presentation

We report our observations of four patients.

Case 1

This is a 72-year-old male married patient admitted at our emergency medicine department (EMD) without any particular pathological history, not known as a smoker, who presented for the past three hours with abrupt onset ptosis of the left eye without an acute tumor syndrome. He also did not present with sensorimotor deficits associated with asthenia worsening at the end of the day, and there were no signs of hypersecretion of the pituitary gland. Total paralysis of the left oculomotor nerve was noted.

**Figure 1:**
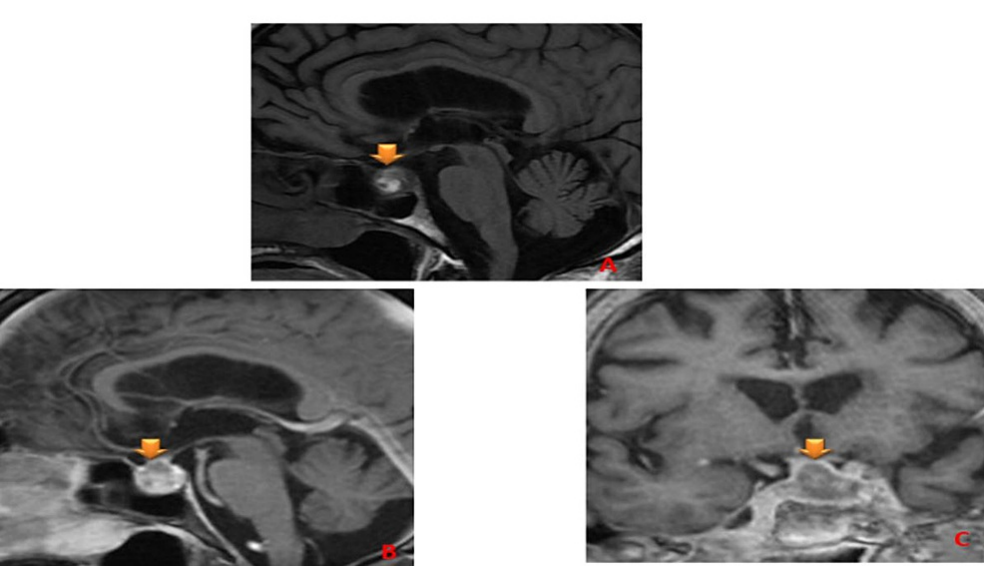
Pre-contrast T1WI and post-contrast T1WI on sagittal (A), (B), and coronal plans (C) demonstrating a sellar and suprasellar heterogeneous lesion with high T1 signal within the mass compatible with acute hemorrhage.

MRI showed a bleeding pituitary macroadenoma and laterosellar macroadenoma (Figure 1).

The hypophysogram demonstrated a lack of corticotropic and thyreotropic functions. The serum levels of cortisol, adrenocorticotropin hormone (ACTH), thyroid-stimulating hormone (TSH), and free thyroxine (FT4) were 3.6 ug/dL, 6 pg/mL, 0.03 uUI/mL, and 8 ng/L, respectively.

The patient had an endoscopic endonasal transsphenoidal resection of the pituitary adenoma. There were no postoperative complications, no polyuria, no polydipsia, and no cerebrospinal fluid rhinorrhea. The patient completely recovered 10 days postoperatively.

The anatomopathological study showed a non-secreting pituitary macroadenoma.

The patient had a remarkable clinical inpatient recovery, with a striking improvement of the ptosis within 10 sessions of the ocular physiotherapy. We kept hormonal replacement therapy including hydrocortisone and levothyroxine.

Case 2

An 87-year-old female widow was admitted at the EMD with a femoral neck fracture, with a significant past medical history of cardiac arrhythmias under anticoagulant therapy and anti-arrhythmic drugs, not known as a smoker.

She presented with anterior hypopituitarism with asthenia and no acute tumor syndrome or pituitary secretion syndrome. However, she mentioned that she was suffering from acute headache episodes for around three months. A hypophysogram was prescribed in her case, which described a global corticotropic, gonadotropic, and thyrotropic insufficiency.

The serum levels of cortisol, adrenocorticotropin hormone (ACTH), follicle-stimulating hormone (FSH), thyroid-stimulating hormone (TSH), and free thyroxine (FT4) were 2 ug/dL, 4 pg/mL, 10 UI/L, 0.05 uUI/mL, and 5 ng/L, respectively.

In her case, we have prescribed levothyroxine and hydrocortisone.

The pituitary MRI objectified a necrotic suprasellar pituitary tumoral growth. Endoscopic endonasal transsphenoidal resection of the pituitary adenoma was contraindicated in her case due to her history of heart rhythm disorder and coagulopathy.

Due to the unavailability of stereotactic radiosurgery (SRS), we opted for conservative treatment. This entailed stabilizing the patient via intravenous rehydration and analgesic therapy while monitoring the patient’s vital signs and neurologic status and hormonal substitution therapy with levothyroxine and hydrocortisone.

After five days of inpatient hospitalization, the patient fully recovered and was discharged under levothyroxine and hydrocortisone prescription and follow-up every month at our outpatient department (OPD).

Case 3

This 82-year-old female married patient presented to our EMD with symptoms of severe cephalalgia and vomiting along with signs of third cranial nerve paralysis. She had a significant past medical history of coagulopathy under anticoagulant therapy, and she was not known as a smoker.

On the other hand, the hypophysogram described corticotropic and gonadotropic deficiency, managed with hydrocortisone. The serum levels of cortisol, adrenocorticotropin hormone (ACTH), and follicle-stimulating hormone (FSH) were 2.8 ug/dL, 5 pg/mL, and 5 UI/L, respectively.

The pituitary MRI showed a hemorrhagic hypophyseal adenoma in line with our diagnosis of pituitary apoplexy.

Conservative management explained in the previous case was preferred for this patient due to her history of coagulopathy, which did not make her a candidate for surgical treatment.

She fully recovered after one week of hospitalization and had a substitution treatment including levothyroxine and hydrocortisone with outpatient follow-up.

Case 4

The patient was a 72-year-old male married patient. He was treated for diabetes and hypertension and is not known as a smoker. He was admitted with febrile consciousness disorders and digestive symptoms including vomiting and diarrhea. He presented ptosis with mydriasis of the left eye. The ophthalmological examination showed no papillary involvement and a narrowing or even non-perception of the isopters in the Goldmann visual field.

The blood tests showed corticotropic and thyroid insufficiency. The serum levels of cortisol, adrenocorticotropin hormone (ACTH), thyroid-stimulating hormone (TSH), and free thyroxine (FT4) were 3 ug/dL, 5 pg/mL, 0.09 uUI/mL, and 4.2 ng/L, respectively.

The MRI results showed an aspect of a pituitary macroadenoma with hemorrhagic infarction corresponding to pituitary apoplexy.

Due to the unavailibility of SRS, the neurosurgery team recommended a conservative treatment explained previously because of the subacute clinical presentation. We supplemented the patient with hydrocortisone and levothyroxine and suggested patient follow-up one month after recovery.

## Discussion

Classical pituitary apoplexy refers to a clinical syndrome defined by sudden onset of frontal or retro-orbital headache, vomiting, visual impairment, and decreased consciousness caused by hemorrhage and/or infarction of the pituitary gland [4]. Apoplexy usually occurs in patients with preexisting pituitary adenomas and evolves within hours or days. Asymptomatic pituitary hemorrhage and/or infarction (“subclinical pituitary apoplexy”) may be detected on routine imaging or during a histopathological examination.

Pituitary adenomas tend to bleed more frequently than other central nervous system tumors [5]. They may bleed or be infarcted or both. It is assumed that large tumors are not well perfused due to insufficient vascularization because of their large size, resulting in necrosis or hemorrhage [6]. Hypertension is the commonest predisposing factor. Major surgery, especially coronary artery bypass grafting, can precipitate apoplexy [7]. Patients undergoing cardiac surgery are at higher risk as a result of fluctuations in blood pressure caused by cardiac bypass and the use of anticoagulant therapy [8]. Pituitary apoplexy should therefore always be considered in patients who develop a headache and neuro-ophthalmic symptoms after major surgery. Dynamic testing of the pituitary gland has been reported to trigger apoplexy [9].

There are also some other factors known to instigate pituitary apoplexies, such as coagulopathies, initiation or withdrawal of dopamine receptor agonists, estrogen therapy, radiation therapy, pregnancy, head trauma, production of tumor intrinsic factor [10], and vasospasm, as well as a systemic arterial pressure system and the arterial supply system of the pituitary gland [11].

It is imperative to take into consideration that the classical presentation of pituitary apoplexy is quite uncommon. However, good clinical skills are necessary to be able to detect this pathology. It is worth mentioning that cases presenting with acute onset of headache and neuro-ocular symptoms should be worked up as a case of apoplexy if there is a high clinical suspicion. It is mainly retro-orbital or frontal and throbbing and is mainly different from the cephalalgias that people describe. The more severe the headache is, the more neuro-ophthalmic signs the patient will present. Other symptoms such as nausea and vomiting may suggest another diagnosis and could delay the diagnosis of apoplexy [12].

The key differential diagnosis of apoplexy includes meningitis, acute hydrocephalus, subarachnoid hemorrhage, hemorrhagic infarction in a Rathke’s cyst, and ischemic cerebral vascular accident. The difference could appear at different sequences of the MRI [13].

There is no consensus in the existing literature about the ideal management for PA [14]; however, studies for practical guidelines showed that the management of PA depends on the time between symptoms and admission, the presence of neurologic deficits, acute deterioration of visual status, and the initial clinical status [15].

If surgical treatment is selected, the procedure would be performed 24 hours after the initial assessment [16]. Surgery is typically selected for patients who presented acute symptoms with no visual deficit.

If apoplexy is suspected, in addition to intravenous fluids, a stress dose of hydrocortisone should be promptly administered intravenously to treat assumed adrenal insufficiency with gradual tapering based on the clinical picture. Upon discharge, an oral maintenance dose of hydrocortisone in two or three divided doses is given until adrenal function is further evaluated [17].

In elderly patients with pituitary apoplexy who have compressive symptoms but for whom surgery is contraindicated, according to global recommendations, if nonsurgical therapy is efficient and the clinical condition does not progress, close monitoring of the patient’s condition and continued conservative treatment may be possible with pleasing outcomes while the possibility of having a full recovery with nonsurgical therapy is very weak.

It is recommended that patients with pituitary apoplexy be managed in a specialized neuroendocrine entity. Surgical therapy, if substantiated, must be realized by a neurosurgeon qualified in neuroendocrine surgery [18].

The UK guidelines for the management of pituitary apoplexy were published in 2010 [19]. The guidelines have a scoring system that could be used as a tool in the follow-up and monitoring of patients who have undergone conservative treatment. This score includes the level of consciousness using the Glasgow Coma Scale, visual acuity, and field defects, as well as ocular paresis. In the majority of cases of pituitary apoplexy during the course of the disease, surgical management is not required as the tumors decrease in size or even disappear without surgical intervention. The outcome of patients with pituitary apoplexy has improved markedly due to novel developments in radiology, surgical skills, and medical technologies.

## Conclusions

Symptomatic pituitary apoplexy is a rare condition. It is nearly always caused by a hemorrhagic or an infarcted pituitary tumor. The most effective management plan for such pathology is early surgical decompression to avoid a mass effect or of the second cranial nerve or chiasma, or of the cranial nerves or cavernous sinuses.

A conservative medical therapy including stabilizing the patient via intravenous rehydration and analgesic therapy, monitoring the patient’s vital signs, and providing hormonal substitution therapy should be indicated if there are no acute symptoms or if the patient is clinically secure for a great amount of time, particularly for elderly patients and those with multiple comorbidities.

Pituitary apoplexy in the elderly typically presents with features of a hypofunctioning pituitary gland, and particularity in older patients, nonspecific symptoms of hypopituitarism may be explained by concomitant pathologies, making clinical assessment difficult.

In our experience, a high index of suspicion is prudent in evaluating an older adult with an unexplained functional decline. Therefore, the patient has to be worked up with a pituitary MRI and hypophysogram blood test.

For the geriatric population with acute clinical symptoms, the probability of performing a clinical remission with a nonsurgical approach is poor. On the other hand, if there are symptoms of compression but surgery is contraindicated and nonsurgical treatment is efficient with a stable clinical state, we could observe the case nearly with close monitoring, and it may have a prompt evolution.
